# Un cas de trichobezoard gastrique

**DOI:** 10.4314/pamj.v9i1.71194

**Published:** 2011-06-17

**Authors:** Mountassir Moujahid, Tarik Ziadi, Issam Ennafae, Hicham Kechna, Omar Ouzzad, Sifeddine El Kandry

**Affiliations:** 1 Service de chirurgie générale, 5^ème^ Hôpital Militaire Guelmim, Maroc

**Keywords:** Trichobézoard, corps étranger intra gastrique, masse gastrique, diagnostic et traitement

## Abstract

Le trichobézoard est défini par la présence d'un corps étranger intra gastrique formé par des cheveux ou des fibres textiles. C'est une pathologie rare qui survient habituellement chez des adolescents présentant des troubles psychiques. La symptomatologie clinique est très variée et le diagnostic est souvent suspecté à la radiologie et à l'endoscopie. Le traitement est chirurgical associé à une prise en charge psychologique. Nous rapportons l'observation d'une jeune femme de 26 ans, hospitalisée au service pour une masse épigastrique. La fibroscopie oesogastroduodénale a mis en évidence un trichobézoard confirmé par le scanner abdominal. La patiente a été opérée par une gastrostomie, les suites post opératoires étaient simples. Elle a été confiée par la suite à un psychiatre pour suivi thérapeutique. Le trichobézoard est une pathologie rare qui survient habituellement chez des adolescents présentant des troubles psychiques.

## Introduction

Le trichobézoard est une affection rare qui survient habituellement chez des adolescents présentant des troubles psychiques. Sa symptomatologie clinique est très variée et le diagnostic est souvent suspecté à la radiologie et à l'endoscopie. Le traitement est essentiellement chirurgical associé à une prise en charge psychologique. Nous rapportons un cas colligé dans le service de chirurgie du 5^ème^ Hôpital Militaire.

## Observation

Il s'agit d'une jeune fille de 26 ans, sans antécédents pathologiques notables ; qui présentait depuis trois mois des douleurs épigastriques à type de crampes et de pesanteurs post prandiales associées à des vomissements alimentaires, des éructations nauséabondes avec une haleine fétide sans trouble du transit intestinal, le tout évoluant dans un contexte d'amaigrissement chiffré à six Kg.

**Figure 1:**
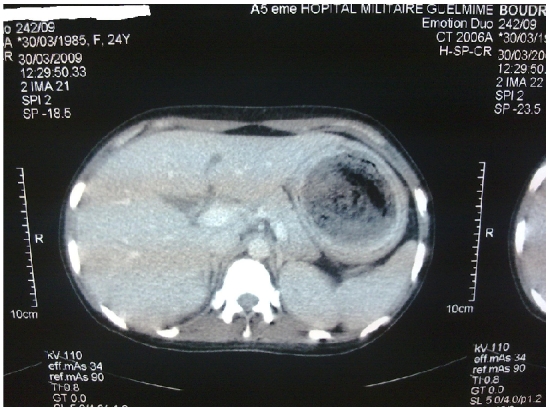
scanner abdominal montrant une image hétérogène de siège intra gastrique chez un patient atteint de trichobezoard gastrique

**Figure 2:**
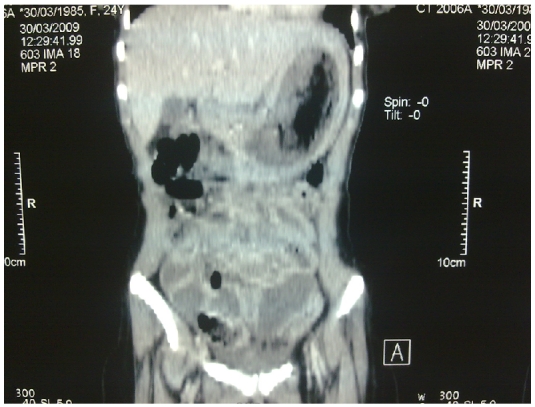
coupe sagittale montrant le trichobézoard gastrique

L'examen clinique a mis en évidence une pâleur conjonctivale modérée avec une masse épigastrique dure, indolore, mobile, de surface lisse. Le reste de l'examen somatique est sans particularité. La fibroscopie oesogastroduodénale a montré une formation gastrique intraluminale faite de cheveux entrelacés mêlés à des aliments occupant tout l'estomac correspondant à un trichobézoard. Le scanner abdominal a mis en évidence une image hétérogène de siège intra gastrique (Figure 1 and 2 et 3).

**Figure 3:**
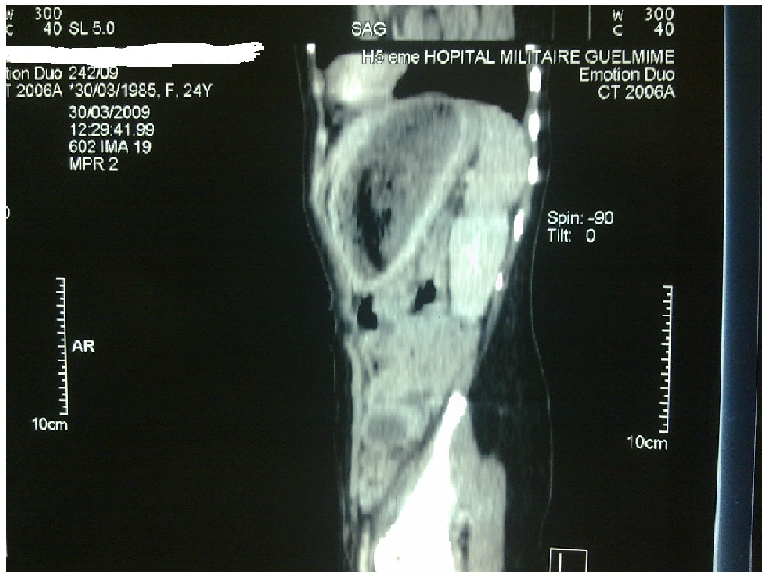
coupe axiale montrant le trichobézoard gastrique

**Figure 4:**
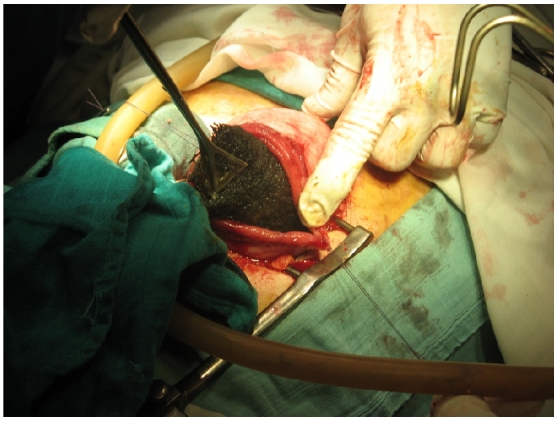
gastrostomie permettant l'extraction du trichobézoard gastrique

Le reste du bilan biologique a mis en évidence une discrète anémie hypochrome avec une hypo protidémie à 60 g/L et une hypo albuminémie à 30 g/L. L′exérèse chirurgicale était alors réalisée à travers une gastrotomie permettant l'extraction d'un énorme trichobézoard épousant toute la forme de l'estomac (Figure 4 et 5).

**Figure 5:**
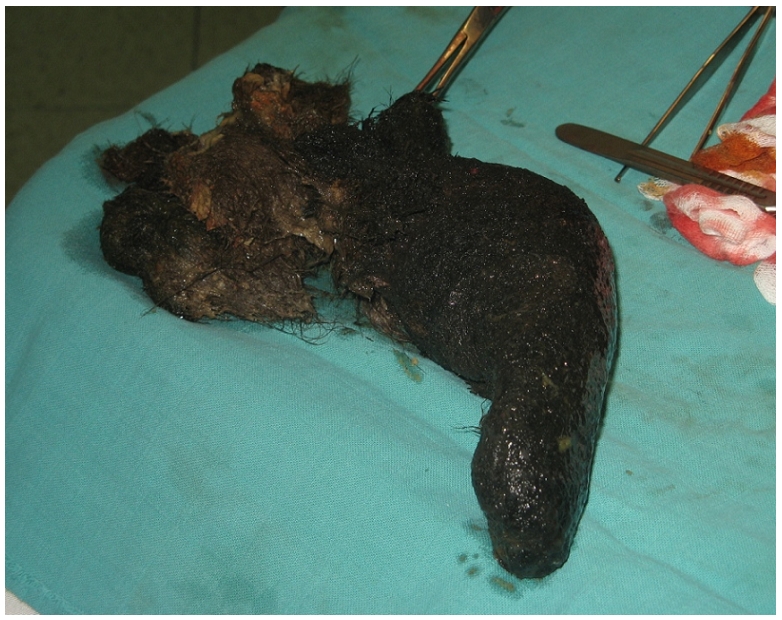
pièce opératoire montrant le trichobézoard gastrique

Les suites postopératoires étaient simples. Une prise en charge psychiatrique était instaurée par la suite chez notre malade.

## Discussion

Le terme “Bézoard” est issu du persan panzehr, ou de l′arabe badzehr, qui signifie antidote ou antipoison [1]. Il désigne une affection rare, secondaire à l′accumulation inhabituelle, sous forme de masses solides ou de concrétions, de substances de diverses natures à l′intérieur du tube digestif et plus particulièrement au niveau de l′estomac, mais aussi parfois dans les voies urinaires. La nature de L'origine des substances détermine le type du bézoard. On distingue le lactéobézoard composé de lait caillé observé chez le nourrisson; le phytobézoard composé de végétaux non digérés et Les trichobézoards qui représentent 55% de tous les bézoards constitués de concrétions de cheveux,de poils ou des fibres de tapis et de débris alimentaires, habituellement confinés dans l′estomac. Exceptionnellement ils peuvent se prolaber dans l′intestin grêle à travers le pylore et être source d'occlusion. D'autres bézoards ont été décrits après la prise de médicaments modifiant le comportement digestif: antiacides, cholestyramine [2]. Le premier cas de trichobézoard a été publié en 1779 [3]. C'est une affection très rare, son diagnostic est clinique et endoscopique. Il est évoqué devant une symptomatologie digestive chronique peu spécifique; surtout chez les jeunes filles ayant des troubles psychiques [1]. Les trichobézoards se forment principalement au niveau de l'estomac, et moins souvent au niveau de l'intestin grêle. Cette affection se manifeste à deux pics d’âge différents. Le premier groupe se situe entre 2 et 6 ans et le second en fin d'adolescence et chez le jeune adulte [2]. Le trichobézoard est observé le plus souvent chez les patients émotionnellement perturbés ou déprimés, les malades psychiatriques, les retardés mentaux et les prisonniers ainsi que chez les patients aux antécédents de chirurgie gastrique, de trichollomanie avec tricophagie [1, 3] .La symptomatologie clinique est très variée type douleurs abdominales, nausées, vomissements, masse abdominale, parfois le bézoard est révélé par une complication telle qu'une hémorragie digestive, une occlusion, une perforation jéjunale [2], ou une pancréatite aiguë imputée à une obstruction de l′ampoule de Vater par un prolongement du trichobézoard réalisant le syndrome de Rapunzel [4].

La fibroscopie oesogastroduodénale reste l′examen de choix en ce qui concerne le diagnostic en permettant la visualisation de cheveux enchevêtrés pathognomonique du trichobézoard. Elle peut, parfois avoir un intérêt thérapeutique en permettant l′extraction endoscopique de petits trichobézoards [1, 3].

L′échographie ne permet de poser le diagnostic que dans 25% des cas, en visualisant une bande superficielle, hyperéchogène, curviligne avec un net cône d′ombre postérieur [4]. La tomodensitométrie avec opacification du tube digestif, ainsi que l'imagerie par résonance magnétique, ont un intérêt moindre dans le diagnostic de trichobézoard. Le traitement est chirurgical permettant l′extraction du trichobézoard gastrique à travers une gastrotomie, ainsi que l′extraction d′éventuels prolongements ou fragments bloqués à distance de l′estomac à travers une ou plusieurs entérotomies [5]. Récemment, la voie laparoscopique a été proposée comme une alternative à la laparotomie [6]. Par ailleurs, une prise en charge psychiatrique, à base de thérapie comportementale, d′éducation parentale et de traitement médical, doit souvent être instaurée chez les patients présentant une trichophagie [1,4,6].

## Conclusion

Le trichobézoard est une pathologie rare qui survient habituellement chez des adolescents présentant des troubles psychiques. La symptomatologie clinique est très variée et le diagnostic est souvent suspecté à la radiologie et à l'endoscopie. Le traitement est chirurgical associé à une prise en charge psychologique.
